# Biomechanical testing of ex vivo porcine tendons following high intensity focused ultrasound thermal ablation

**DOI:** 10.1371/journal.pone.0302778

**Published:** 2024-05-07

**Authors:** William Chu Kwan, Ari Partanen, Unni Narayanan, Adam C. Waspe, James M. Drake

**Affiliations:** 1 The Hospital for Sick Children, Toronto, Ontario, Canada; 2 Profound Medical, Mississauga, Ontario, Canada; University College London Institute of Child Health, UNITED KINGDOM

## Abstract

**Introduction:**

Magnetic resonance-guided focused ultrasound (MRgFUS) has been demonstrated to be able to thermally ablate tendons with the aim to non-invasively disrupt tendon contractures in the clinical setting. However, the biomechanical changes of tendons permitting this disrupting is poorly understood. We aim to obtain a dose-dependent biomechanical response of tendons following magnetic resonance-guided focused ultrasound (MRgFUS) thermal ablation.

**Methods:**

Ex vivo porcine tendons (n = 72) were embedded in an agar phantom and randomly assigned to 12 groups based on MRgFUS treatment. The treatment time was 10, 20, or 30s, and the applied acoustic power was 25, 50, 75, or 100W. Following each MRgFUS treatment, tendons underwent biomechanical tensile testing on an Instron machine, which calculated stress-strain curves during tendon elongation. Rupture rate, maximum treatment temperature, Young’s modulus and ultimate strength were analyzed for each treatment energy.

**Results:**

The study revealed a dose-dependent response, with tendons rupturing in over 50% of cases when energy delivery exceeded 1000J and 100% disruption at energy levels beyond 2000J. The achieved temperatures during MRgFUS were directly proportional to energy delivery. The highest recorded temperature was 56.8°C ± 9.34 (3000J), while the lowest recorded temperate was 18.6°C ± 0.6 (control). The Young’s modulus was highest in the control group (47.3 MPa ± 6.5) and lowest in the 3000J group (13.2 MPa ± 5.9). There was no statistically significant difference in ultimate strength between treatment groups.

**Conclusion:**

This study establishes crucial thresholds for reliable and repeatable disruption of tendons, laying the groundwork for future in vivo optimization. The findings prompt further exploration of MRgFUS as a non-invasive modality for tendon disruption, offering hope for improved outcomes in patients with musculotendinous contractures.

## Introduction

Musculotendinous contractures in skeletal muscles and tendons lead to a reduction in the range of motion (ROM) in a joint, and are associated with impairment of activities of daily living, mobility, independence, and increased pain and disability. [1–3] Musculotendinous contractures are associated with pediatric conditions such as cerebral palsy, neuromuscular disorders, and clubfoot, among others. It is frequently observed in adults with traumatic brain injury, spinal cord injury, or following a stroke. [4–7] The high incidence is usually described in the literature in relation to specific disorders. [1–4] In children with cerebral palsy, it is reported that 36% have upper extremity contractures, 69% have reduced hand control, [8] and 22% have knee contractures. [9] In the institutionalized elderly, the reported incidence ranges from 20 to 80% depending on the studies. [10, 11]

Non-surgical treatment of contractures, such as splinting or serial casting, are lengthy and often ineffective. [12–17] Surgical options are associated with significant morbidity, costs, often requiring extensive rehabilitation, and could be associated with recurrence, which would require re-treatment [18–23]. Numerous surgical procedures for musculotendinous lengthening have been described in the literature [18–21]. They are performed under general anesthesia with incisions of several centimeters and often require extensive soft tissue dissection depending on the tendon affected. Lengthening is achieved by partial or complete resection, weakening the tendinous structure, allowing for desired elongation of the tendon which leads to an increase in ROM. If the ROM is suboptimal, further tendon resection is repeated until a desired ROM is achieved. As in any surgical procedure, patients are exposed to complications including delayed wound healing, infection, incisional pain, and systemic effects such as urinary retention, blood clots, and pneumonia increasing the need for hospitalization and post-surgical care. Surgical procedures require anesthesia, intubation, skin incision, prophylactic antibiotic, and soft-tissue dissection, which could compromise neurovascular structures. These complications are exacerbated in patients with poor baselines and other medical comorbidities [22]. Outcomes of surgical lengthening gives relief to musculotendinous contractures in the short and long-term, demonstrating an increase of the affected joint angle, and less pain [4, 22].

High-intensity focused ultrasound (HIFU) is a non-invasive ultrasound therapy modality that uses extracorporeally placed transducers that focus sound waves within the body. The transducer emits multiple sound waves that penetrate and travel through tissue layers with minimal energy loss and constructively add at a focal point deep in the body [24, 25]. A continuous application of sound waves at the focal point results in energy absorption leading to an increase in temperature and subsequent tissue ablation [24–26], At the cellular level, organelle disruption and protein denaturation lead to therapeutic tissue necrosis [27, 28]. When HIFU is coupled with magnetic resonance imaging (MRI) to guide, monitor and evaluate its treatment accuracy, it is referred to as magnetic resonance-guided focused ultrasound (MRgFUS). To ensure that unintended hot spots are not being produced, treatments are monitored using proton resonance frequency shift MR thermometry (PRFS-MRT), which measures the change in temperature of heated water molecules and produces dynamic thermal maps with an uncertainty of < 1°C [29]. The non-ionizing nature of MRgFUS allows for repeat treatments to be safely performed without long-term sequelae [30]. MRgFUS is clinically approved for bone metastasis, soft-tissue tumors, uterine fibroids and essential tremor [31, 32]. MRgFUS has been shown to disrupt in vivo porcine tendons and has the potential to be used a possible non-invasive treatment for musculotendinous contracture [33]. We hypothesize that thermal ablation of tendons denatures the protein fibers and changes its biomechanical properties facilitating disruption of ablated tendons. This paper aims to understand the biomechanical impact of MRgFUS ablation on tendons to quantitative the amount of force required to rupture a tendon both before and after MRgFUS ablation and provide further evidence that this bioeffect changes the modulus of elasticity and ultimate tensile strength of the tendon following MRgFUS ablation. This dose-response characterization would guide future parametric study for application in the clinical setting.

## Materials and methodology

### Tendon phantom preparation

**Fig 1:**
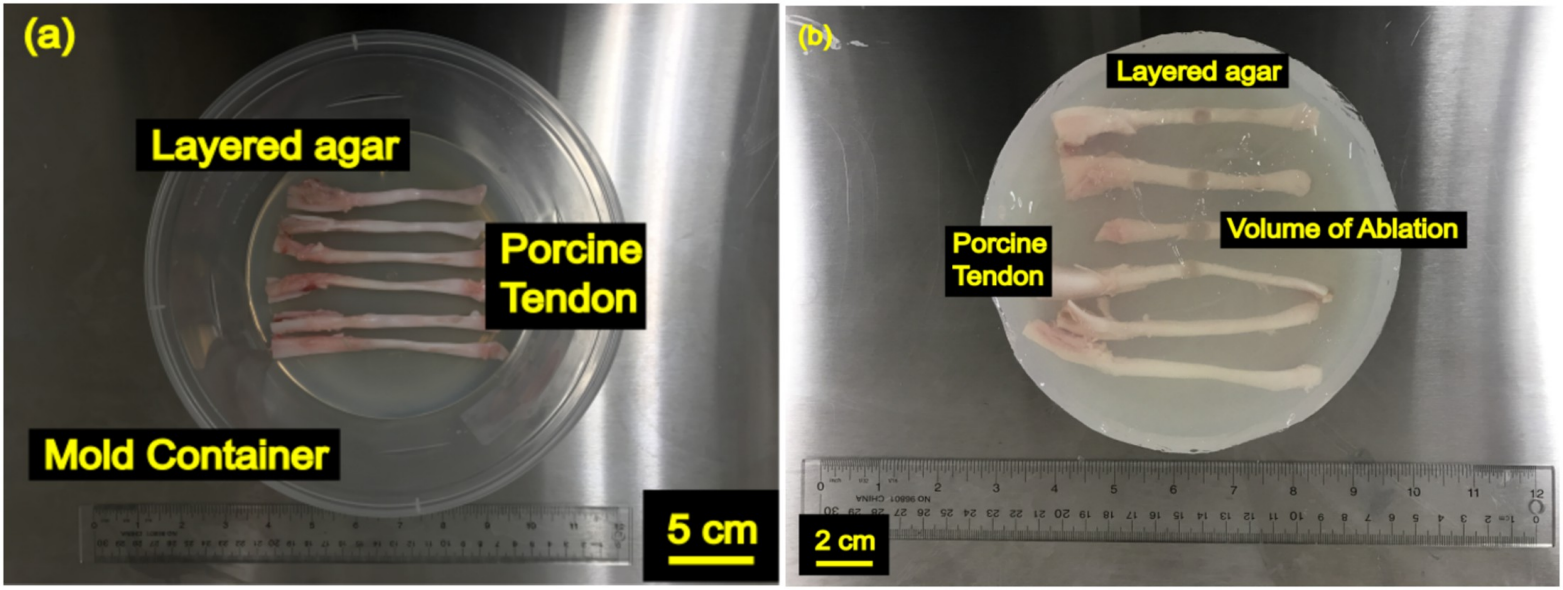
(a) Experimental Setup for *ex vivo* porcine tendon in layered agar prepared in a mold. (b) Tendon following treatment with MRgFUS sonication with ablation marks.

All animal procedures were approved by the Animal Care Committee at the Hospital for Sick Children (Toronto, Ontario, Canada) under AUP47783. Fresh 16-week porcine plantar digital tendons from 16-week old pigs (n = 72) were dissected from pig feet purchased from a local abattoir and extracted within 8 hours [34]. A digital caliper was used to measure the long and short perpendicular axis diameter of tendons given their elliptical cross-sectional shape. The cross-sectional area was calculated using these diameters. The tendons underwent vacuum degassing in saline for one hour [35, 36]. High gel strength agar (2% w/v) was dissolved in aqueous solvent to a temperature of 95°C. When the temperature of the solution was lowered to 37°C, a first pour in a mold provided a bed of 2 cm of thickness for the tendons to be embedded in. Next, the degassed tendon was placed on this agar bed, and a second pour of 2 cm embedded the tendons in solid agar phantom with care not to entrap any bubbles during each pour [37]. The agar phantom with tendons embedded were placed in a fridge at 4°C and stored overnight and brought back to room temperature (20°C) prior to HIFU treatment as shown in Fig 1a [38].

### MRgFUS setup and treatment

A 3T Achieva MRI system (Philips Healthcare, Best, the Netherlands) and a Sonalleve three channel pelvic coil were used for imaging. Acoustic coupling was achieved using reversed osmosis degassed water with a 3.5mm ultrasound gel pad (Aquaflex, Parker Laboratories, Fairfield, NJ, USA). Coupling quality was verified using a bubble scan to ensure reduced ultrasound wave propagation interference, avoid near-field attenuation and reflection of ultrasound waves. Next, a bubble scan of the tendon phantom was performed to ensure minimal attenuation of the ultrasound path. T2-w MRI sequences were used for MRgFUS planning and verification after sonication as the short T2 in tendon produces strong contrast between the tendon and phantom. Real-time thermometry, scaled from 20°C to 37°C to account for Sonalleve system limitations, using the proton resonance frequency shift MR thermometry (PRFS-MRT) sequence has a temperature uncertainty of less than 1°C and a spatial accuracy of 1.5 x 1.5 x 5.5 mm. [39–42] It is an FDA-approved sequence for clinical temperature mapping and has been validated by other groups [39, 43]. The pulse sequences are summarized in Table 1.

**Table 1:** Summary of MRI and PRF-based MRT pulse sequence parameters. (FOV = field of view. TE = Time to Echo. TR = Repetition Time. NSA = Number of Excitations. FFE = Fast Field Echo. EPI = Echo planar Imaging).

	Bubble Scan	Treatment Planning	MR Thermometry
**Sequence**	T1 3D FFE	T2 3D FFE	4 plane FFE-EPI
**FOV (mm)**	280 x 280 x 36	250 x 250 x 50	168 x 168 x 5.5
**Thickness (mm)**	2	1	5.5
**Resolution (mm)**	1.09 x 1.1 x 2	1x1x1	1.5 x 1.5 x 5.5
**No. Slices**	18	50	1/plane
**TE (ms)**	12	12	16
**TR (ms)**	15	15	26
**NSA**	1	1	1
**Flip angle (degree)**	10	20	19.5

**Fig 2:**
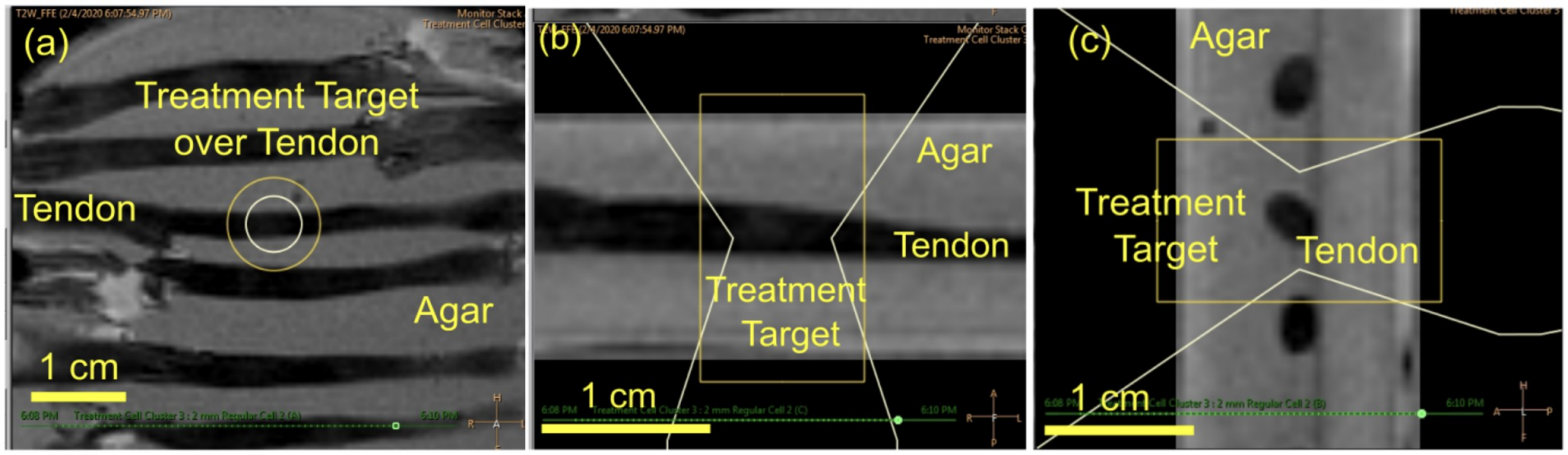
T2-w MRI scan of tendons in agar mold and MRgFUS treatment target focused on treatment target for (a) coronal, (b) transverse, and (c) sagittal planes.

**Fig 3:**
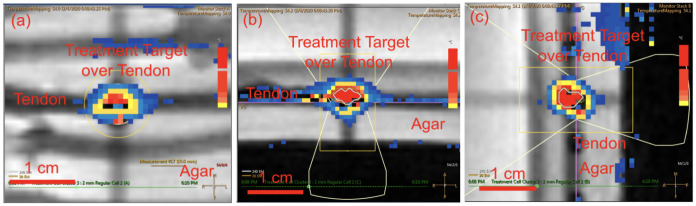
MR thermometry during the last second of MRgFUS treatment displaying a temperate map of MRgFUS tendon ablation for (a) coronal, (b) transverse, and (c) sagittal planes.

**Fig 4:**
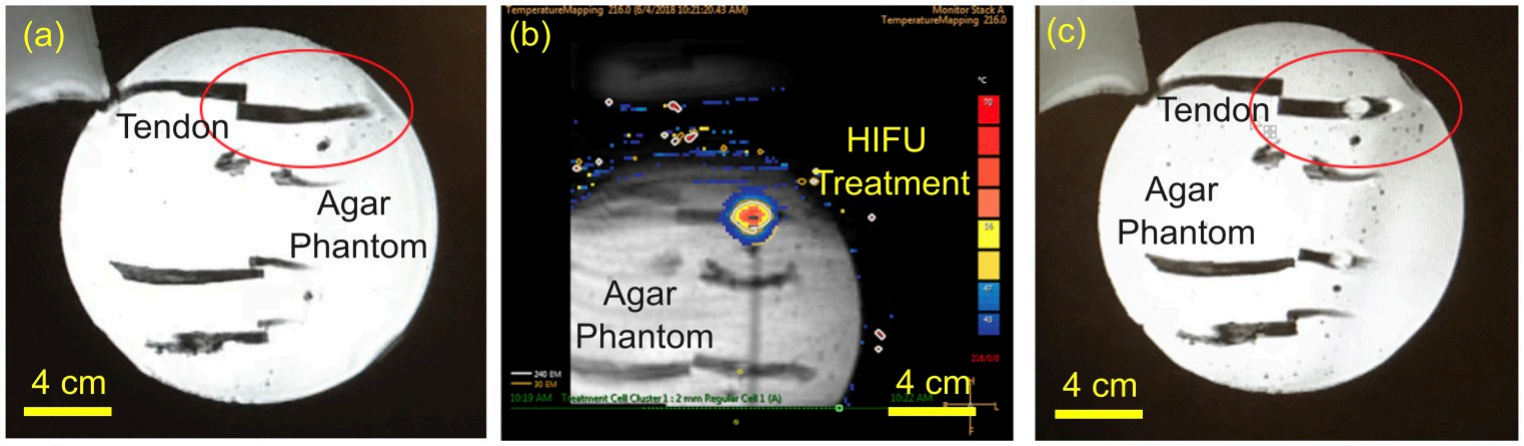
Coronal views of MRgFUS targeted tendon (a) T2-MRI prior to MRgFUS, (b) during MR thermometry, and (c) following MRgFUS ablation showing hyperintense area.

HIFU sonications were conducted using a Sonalleve V1 MRgFUS system (Profound Medical Inc., Ontario, Canada) with a transducer frequency = 1.2MHz, number of elements = 256, surface diameter = 128mm, radius of curvature = 120mm, and focal spot size of 1.5 x 1.5 x 9.2 mm (assuming linear wave propagation) [44]. Baseline temperature of the setup was monitored using a fiberoptic probe. A total of 78 tendons were randomly assigned to one of 12 treatments groups and a control group in a dose response experimental design based on *a priori* sample size analysis for binary outcome rupture rate (p1 = 0.05, p2 = 0.95, α = 0.05, power = 0.80) requiring a sample size of 5 per group with an additional control tendon added to each gel pad [45]. The treatment groups were defined by permutations of ablation powers (25W, 50W, 75W, and 100W) combined with ablation time (10s, 20s, and 30s) based on the energy requirements for MRgFUS treatment for other musculoskeletal targets such as osteoid osteoma [46]. An MRgFUS treatment cell was placed in the center of the cross-section of each tendon as shown in the T2-w MRI in Fig 2. Real-time temperature scans were performed during HIFU sonications as shown in Figs 3 and 4 show T2-w MRI before and after MRgFUS.

### Instron testing

**Fig 5:**
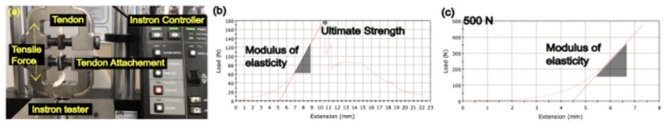
(a) Instron tensile test setup (b) load-extension curves for tendons that had enough energy for rupture, and (c) load-extension curves for tendons that did not rupture with maximal load of 500N corresponding to the limit of the system.

Following MRgFUS ablation, tendons were removed from the gel pad and examined for visual signs of thermal ablation. Next, biomechanical testing was performed using an Instron Universal Testing System (2710–112 Norwood, MA, USA) with maximum load of 500N and maximum torque of 2N-m. The tendons were secured using a combination of serrated jaws with gritted sand paper #24 grit as shown in Fig 5a. The distance between the upper and lower jaws was set to 1cm outside of the HIFU treatment affected area and verified using a digital caliper. A tensile force was applied to deform until rupture was reached or the maximal tensile force of 500N was applied. Stress-strain curves are automatically displayed by the Instron system, which were used for detailed biomechanical testing analysis.

### Statistical analysis

The primary endpoint was tendon disruption during Instron testing. A bias reduction in binomial-response generalized regression models was performed to elucidate the predominant factor to predict tendon rupture. Factors considered were power, time, and energy (multiplicative power and time). Tendon cross-sectional area was calculated; an ANOVA test and a Tukey’s post-hoc test was used to verify any statistical difference between in each treatment group. The maximum temperatures achieved during each treatment was defined as the average of the most significant 3x3 pixel centered over the target cell. The average maximum temperatures were tested for homogeneity with a Levene test and were compared using an ANOVA test and a Tukey’s post-hoc test was used to verify any statistical difference in each treatment group. Modulus of elasticity was calculated as the slope of the load-extension curve. The ultimate strength was measured as the highest load prior to failure in the load-extension curve as show in Fig 5b and 5c. All biomechanical parameters underwent homogeneity test with a Levene test and were compared using an ANOVA test and a Tukey’s post-hoc test was used to verify any statistical difference in each treatment group.

## Results

### Tendon rupture

A total of 6 tendons were allocated for each of the 12 treatment groups and a control group. There was no statistically significant difference in the dimensions of tendons. Table 2 shows the number and percentage of tendons disrupted following each treatment group which factors in sonication power and time. As energy represents the power delivered over a period of time, Table 3 summarizes tendons disrupted by energy delivered. The remainder of this study will present all results in terms of energy. There was an increasing trend in rupture rate as energy was increased. Table 3 summarizes each of the regressions performed with the statistically significant factor with comparable Akaike information criterion (AIC) being power alone (p < 0.00001), and energy alone (p < 0.00001), while time alone was not a statistically significant factor [47].

**Table 2:** Frequency table for tendon rupture for each of the 12 treatment groups, which combines ablation power and treatment time.

	25W	50W	75W	100W
**10s**	0 (0%)	0 (0%)	2 (33%)	5 (83%)
**20s**	1 (16%)	1 (16%)	3 (50%)	6 (100%)
**30s**	0 (0%)	2 (33%)	6 (100%)	6 (100%)

**Table 3:** Statistical significance of regression using power and time as factors affecting rupture.

Regression	P-value	AIC
**Power**	<0.00001	60.296
**Time**	0.0527	98.731
Energy = Power * Time	<0.00001	60.255

### Maximum temperature by group

**Fig 6:**
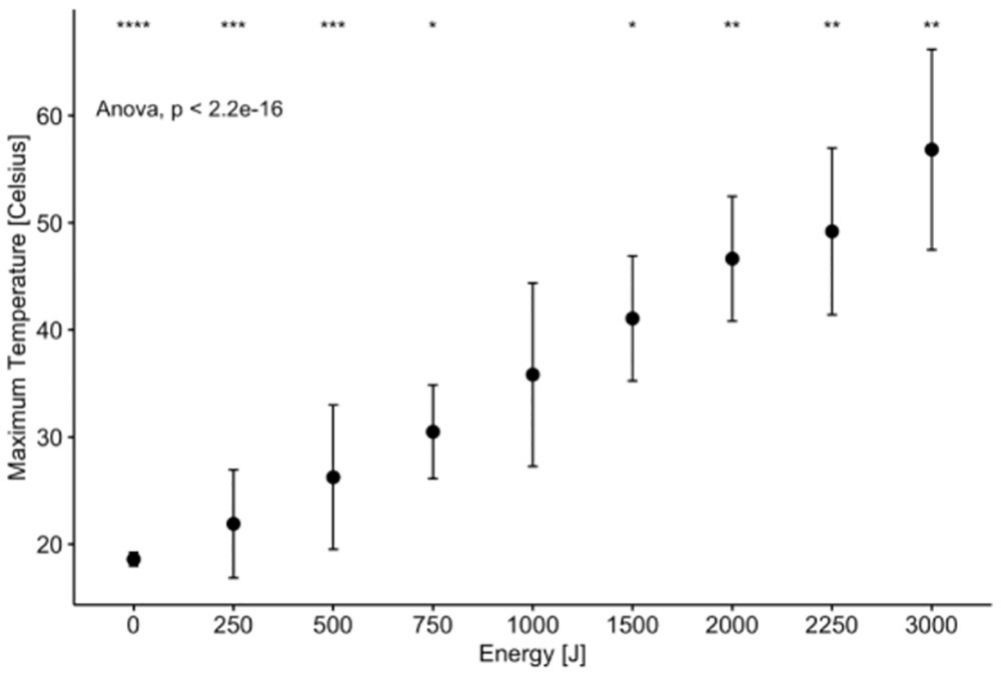
Graph plotting maximum temperatures of treatment as a function of applied energy, including 0J control.

The maximum temperature achieved during sonication was analyzed and summarized on Tables 4 and 5, and Fig 6. The highest recorded temperature of 56.82°C +/- 9.35 was at the highest energy delivered (3000J), while the lowest recorded temperature of 18.60°C ± 0.63 was for the control group, which did not register an increase in temperature. An ANOVA test and a Tukey’s post-hoc test showed a statistically significant increase in temperature between most energy treatments.

**Table 4:** Average maximum temperature during sonication for each treatment group.

	25W	50W	75W	100W
**10s**	21.9 ± 5.0	26.5 ± 5.6	31.7 ± 5.7	34.1 ± 7.8
**20s**	26.0 ± 8.2	37.2 ± 9.7	44.2 ± 5.6	46.7 ± 5.8
**30s**	29.3 ± 2.3	38.0 ± 4.5	49.2 ± 7.8	56.8 ± 9.4

**Table 5:** Summary of tendon disruption and average maximum temperature by treatment energy delivered.

Energy	Tendon	Rupture	Max Temp
**0 J (Control)**	0	0 (0%)	18.6 ± 0.6
**250 J**	6	0 (0%)	21.9 ± 5.1
**500 J**	12	1 (8.3%)	26.3 ± 6.7
**750 J**	12	2 (16.7%)	30.5 ± 4.4
**1000 J**	12	6 (50%)	35.8 ± 8.6
**1500 J**	12	5 (41.7%)	41.1 ± 5.8
**2000 J**	6	6 (100%)	46.7 ± 5.8
**2250 J**	6	6 (100%)	49.2 ± 7.8
**3000 J**	6	6 (100%)	56.8 ± 9.4

### Instron testing

**Fig 7:**
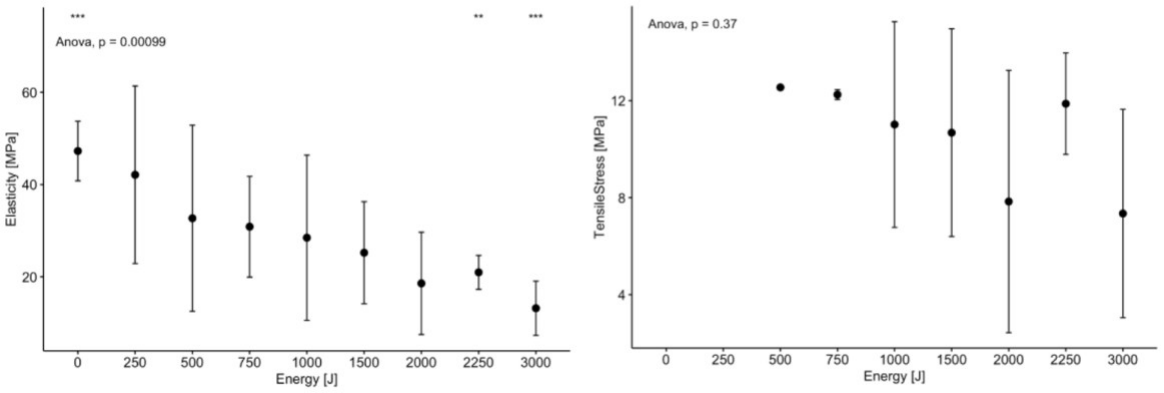
Graph plotting (a) elasticity, and (b) tensile strength measuring during biomechanical testing for tendon disruption as a function of applied energy including 0J control.

During biomechanical testing, all cases of tendon rupture occurred in the midsection of the stretched tendon and not on the clamp-tendon interface. The modulus of elasticity was highest in the control group (47.25 MPa ± 6.46) and lowest in the 3000J group (13.18 MPa ± 5.87) with statistical significance measured between control (0J) and 2000J, 2250J, and 3000J groups, as shown in Table 6 and Fig 7. The ultimate strength, analyzed from each load-extension curve obtained, is summarized in Table 6 and Fig 7. There was not tendon rupture in the control (0 J) and 250 J groups. There was no statistically significant difference between each energy group.

**Table 6:** Summary of biomechanical testing for tendon disruption for energy groups.

Energy	Elasticity [MPa]	Ultimate Strength [MPa]
**0 J (Control)**	47.3 ± 6.5	No failure
**250 J**	42.1 ± 19.2	No failure
**500 J**	32.7 ± 20.2	12.5 (N = 1)
**750 J**	30.9 ± 10.9	12.2 ± 0.2
**1000 J**	28.5 ± 17.9	11.0 ± 4.2
**1500 J**	25.2 ± 11.1	10.7 ± 4.3
**2000 J**	18.6 ± 11.1	7.8 ± 5.4
**2250 J**	21.0 ± 3.7	11.9 ± 2.1
**3000 J**	13.2 ± 5.9	7.4 ± 4.3

## Discussion

Our study performed a dose response characterization of the quantitative changes in tendon biomechanics following MRgFUS ablation in ex vivo porcine tendons. The tendons were embedded in agar phantoms as it matches the physical properties of tissue with an attenuation of 2.01 Np/m, heat capacity of 0.6 W/mK, and thermal conductivity of 4120 J/kgK.[48–50]. A continuous wave 1.2MHz focused treatment with durations of 10, 20, or 30s, for powers of 25, 50, 75, or 100W, delivered the following energies: 250, 500, 750, 1000, 1500, 2000, 2250, and 3000J. With the aim of non-invasive MRgFUS tendons disruption for contracture, it was necessary to evaluate the ability to disrupt tendon in the clinical setting, in which a health professional would have to push on the joint of the disrupted tendon after treatment. Tendons underwent biomechanical Instron testing with maximum load of 500N, beyond the limit of the human push strength of 250N [51].

In our study, when more than 1000J of energy is delivered, over 50% of tendons can be disrupted with up to 100% of tendons disrupted using 2000J, 2250J and 3000J energy doses. The maximum temperature achieved during each MRgFUS treatment was proportional to the energy delivered, with the highest recorded temperature (56.82°C +/- 9.35) during a 3000J sonication and the lowest recorded temperature (18.60°C ± 0.63) in the control group. A similar inversely proportional trend was seen in the Young’s modulus, with the highest in the control group (47.25 MPa ± 6.46) and lowest in the 3000J group (13.18 MPa ± 5.87). Finally, there was no statistical significance in the ultimate strength between the groups where rupture was observed.

Ex vivo animal studies have reported a wide range of biomechanical properties for animal tendons. Young’s modulus values range from 1 MPa (rat tail) to 1600 MPa (porcine plantar digital tendons) [52, 53]. Ultimate strength values range from 2.60 MPa (dog infraspinatus) to 128 MPa (horse common digital extensor tendon) [54, 55]. Three articles have investigated biomechanical properties for porcine plantar digital tendons, but only one value was reported for Young’s modulus (1600 MPa) and one value for ultimate strength (80 MPa) [53, 56, 57]. In a literature review, it is concluded that ex vivo animal tendons, despite common features, show remarkable variations in molecular and biomechanical properties [58]. Hence, the focus of this study is not on the absolute numbers but on the trends of MRgFUS treatment.

Previous studies have investigated high intensity focused ultrasound (HIFU) in tendons. Chu-Kwan et. al studied HIFU ablation in in vivo porcine tendon for contracture [33]. In this study, in vivo tendons were randomized to treatments of 600, 900, 1200 and 1500 J and reported rupture rates of 29%, 86%, 100% and 100% with maximum temperatures of 58.4°C, 63.3°C, 67.6°C, and 69.9°C, respectively. We observe a trends of increasing rupture rate and increasing temperature with increase in energy in both studies. Muratore et. al studied HIFU ablation in ex vivo bovine tendon for the possible treatment of tendinosis [59]. Smallcomb et al. studied HIFU histotripsy in ex vivo rat tendon disruption for possible treatment of chronic tendinopathies [60, 61]. Hazlewood et al. studied pulsed HIFU, not ablation, for in vivo tendon contracture [62]. There has been no studies aiming to investigate the biomechanical changes of tendons following HIFU ablation despite thousands of new papers published in the last ten years that involve biomechanics of tendons [63].

The results presented should be taken in the context of this study. Given the large parametric time and power study, the agar phantom was designed to maximize the number of tendons to be independently treated within the workspace of the Sonalleve although it did not replicate the anatomy, and mechanical properties of a limb. Ex vivo experimentation does not allow to study the inflammatory responses and tissue healing in an in vivo animal model. However, as a preliminary dose response study, it has demonstrated thresholds for HIFU ablation for reliable and repeatable disruption of ex vivo animal tendons. More than 50% of tendons were disrupted when more than 1000J of energy was applied in the treatment. 100% of tendons were disrupted when more than 2000J of energy was applied, providing the foundation to narrow the parameters for future in vivo studies. Hence, this ex vivo study reduces the number of future live animal experiments following the replacement, reduction and refinement principles [64]. Another limitation is the usage of healthy tendons when the future studies aim to study tendon contracture. However, human tendon biomechanical parameters show great variability, depending on age, level of exercise, anatomical site, and steroid use [65, 66]. Given this variability and lack of concluding data, the use of our healthy porcine models is appropriate.

## Conclusion

This study aimed to determine to characterize the quantitative changes in tendon biomechanics properties following MRgFUS ablation in ex vivo porcine tendons. We found that as more energy is delivered to the system, the higher the temperature achieved leading to a decreased in Young’s modulus. This decrease makes it feasible to mechanically disrupt tendons, which has the potential for clinical applications in musculotendinous contractures as non-invasive modality to lengthening tendons to increase the range of motion in affected joint angle [67, 68]. Many frail patients suffering from contractures are not surgical candidates. They do not undergo surgery despite potentially benefiting from a tendon lengthening. Our research is the foundation for further characterization of MRgFUS ablation as a possible modality for tendon disruption.
